# Normalized spatial complexity analysis of neural signals

**DOI:** 10.1038/s41598-018-26329-0

**Published:** 2018-05-21

**Authors:** Huibin Jia, Yanwei Li, Dongchuan Yu

**Affiliations:** 1 Key Laboratory of Child Development and Learning Science (Ministry of Education), Research Center for Learning Science, School of Biological Sciences & Medical Engineering, Southeast University, Nanjing, Jiangsu China; 2 College of Preschool Education, Nanjing Xiaozhuang University, Nanjing, Jiangsu China

## Abstract

The spatial complexity of neural signals, which was traditionally quantified by omega complexity, varies inversely with the global functional connectivity level across distinct region-of-interests, thus provides a novel approach in functional connectivity analysis. However, the measures in omega complexity are sensitive to the number of neural time-series. Here, normalized spatial complexity was suggested to overcome the above limitation, and was verified by the functional near-infrared spectroscopy (fNIRS) data from a previous published autism spectrum disorder (ASD) research. By this new method, several conclusions consistent with traditional approaches on the pathological mechanisms of ASD were found, i.e., the prefrontal cortex made a major contribution to the hypo-connectivity of young children with ASD. Moreover, some novel findings were also detected (e.g., significantly higher normalized regional spatial complexities of bilateral prefrontal cortices and the variability of normalized local complexity differential of right temporal lobe, and the regional differences of measures in normalized regional spatial complexity), which could not be successfully detected via traditional approaches. These results confirmed the value of this novel approach, and extended the methodology system of functional connectivity. This novel technique could be applied to the neural signal of other neuroimaging techniques and other neurological and cognitive conditions.

## Introduction

The human brain is a complex and dynamic system of functional connected regions. The functional connectivity, which is defined as the statistical interdependence or synchronization between neural signals of spatially remote brain areas and could be quantified by various power-based metrics or phase-based metrics, is thus crucial to elucidating how neurons and neural networks process information^1–4^. Analysis of the time-averaged or dynamic functional connectivity metrics, which were derived from brain signals recorded by functional magnetic resonance imaging (fMRI), functional near-infrared spectroscopy (fNIRS), electroencephalography (EEG) or magnetoencephalography (MEG), revealed that large-scale and coherent brain networks were modulated by various psychiatric disorders and cognitive processes, and exhibited temporal dynamics on sub-second timescales^2,5–10^.

The spatial complexity of neural signals in region-of-interests (ROIs) is defined as the heterogeneity of neural signals in different ROIs and varies inversely with the global functional connectivity level across these ROIs, thus provides an alternative technique beyond commonly used approaches in functional connectivity analysis, e.g., independent component analysis (ICA), seed-based/ROI-based approach and graph theory based network analysis^11–13^. The omega complexity, proposed by Wackermann (1996), could be considered as an indicator of spatial complexity, and is calculated as “Shannon entropy of the eigenspectrum of the covariance matrix of neural signals”^14,15^. If neural activities of these ROIs are perfectly synchronized, the omega complexity is lowest (i.e., the omega complexity is equal to 1 in this case). However, if these neural activities are completely independent between each other, the omega complexity is highest (i.e., the omega complexity is equal to the number of ROIs in this case). In this approach, several measures, including global spatial complexity (GSC), regional spatial complexity (RSC) and local complexity differential (LCD), have been developed^16^. The GSC and RSC could be used to quantify the global functional connectivity level or spatial complexity across all ROIs and across all elements within a given ROI, respectively^14,17,18^. The term *element* may refer to “voxel” in fMRI studies, and “channel” in EEG and MEG studies. The LCD provided an index to estimate the contribution of activities of specific ROI to the global functional connectivity of all ROIs^19^.

Although the measures in omega complexity analysis were found sensitive to different types of cognitive processes, chronological ages, neuroactive substances and neuropathological variables^12,13,17,18,20,21^, an important limitation should be mentioned, i.e., the measures in this approach are sensitive to the number of neural processes defined. This makes us could not statistically test whether the regional spatial complexity of a ROI is significantly different from that of the other ROI, when the number of elements is different between these two ROIs. Moreover, since LCD of a specific ROI is estimated by the variation of spatial complexity obtained by excluding this ROI from the computation of spatial complexity, it would also be significantly influenced by the above limitation. Thus, an alternative approach is highly needed.

The techniques used in spatial complexity analysis or functional connectivity analysis could be used to probe into the neurophysiological mechanism of Autism Spectrum Disorder (ASD), which describes a range of polygenetic developmental disorders that are characterized by impairments in social communicative development, along with repetitive stereotyped behaviors and/or restricted interests^22,23^. Increasing evidence supported the theory that altered neural connectivity is a key neural underpinning of the brain of ASD individuals^24–28^. Most of related studies estimated functional connectivity metrics through brain signals recorded using fMRI, EEG or MEG^4,29–31^. However, these neuroimaging techniques are not well suitable for ASD children research, since they are sensitive to head movement of these children. FNIRS, which capitalizes on the physical principle that infrared light scattering over brain tissues reflects ongoing changes in oxygenation/deoxygenation levels, is a promising non-invasive brain imaging technique to investigate the neurodevelopment of young children with ASD, since it’s safe, portable, low cost, and relatively insensitive to head movement^5^. For example, an fNIRS study in our group found that the lobe-level inter-region connections between the right prefrontal cortex and other brain regions (e.g., the left prefrontal cortex and the bilateral temporal lobe) were significantly weaker in young children (younger than 8 years old) with ASD, compared with age- and gender-matched typically developing (TD) children^32^.

In the current study, normalized spatial complexity analysis was proposed, which could overcome the limitations inherent in traditional omega complexity. In order to valid the value of this approach, the neuropathological mechanisms of ASD were investigated using the novel metrics derived from normalized spatial complexity, which provided results that were consistent with and extended findings using traditional functional connectivity analysis and omega complexity analysis. Specially, we hypothesized that abnormalities in the right prefrontal cortex could also be observed through normalized spatial complexity analysis, which was consistent with previous studies^26,32^. Moreover, some novel results could also be detected, suggesting additional information about the brain development of young children with ASD beyond the traditional omega complexity analysis and functional connectivity analysis.

## Results

### Time-averaged normalized spatial complexity

Significant group difference between the two groups was detected for nGSC (*t* = 2.28, *df* = 22, *p* = 0.03 < 0.05). The nGSC of ASD group (0.56 ± 0.05) was significantly larger than that of TD group (0.52 ± 0.03).

**Figure 1:**
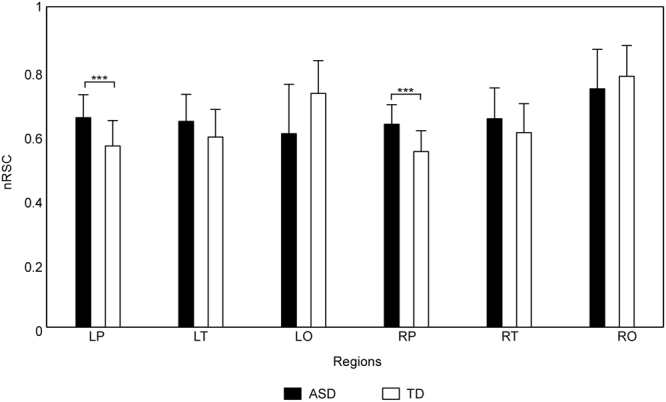
The statistical results of nRSCs. Means and standard deviations of nRSCs of six brain regions (i.e., LP, LT, LO, RP, RT and RO) for each group (ASD group: black bar; TD group: white bar), with error bars representing the standard deviations. Group differences marked by *** are corresponding to the significance level 0.001 (FDR-corrected).

For the nRSCs of six brain regions (i.e., LP-nRSC, LT-nRSC, LO-nRSC, RP-nRSC, RT-nRSC and RO-nRSC), the main effect of brain region and the interaction between brain region and group were significant (*F* (5, 110) = 12.03, *p* < 0.001, and *F* (5, 110) = 5.35, *p* < 0.01 respectively). Further analysis showed that (1) the nRSCs of bilateral prefrontal cortices were significantly smaller than those of bilateral temporal lobes, and the nRSCs of bilateral temporal lobes were significantly smaller than those of bilateral occipital lobes; (2) as has been shown in Fig. 1, the LP-nRSC and RP-nRSC of ASD group were significantly larger than those of TD group. The main effect of group was not significant (*F* (1, 22) = 0.65, *p* > 0.05).

**Figure 2:**
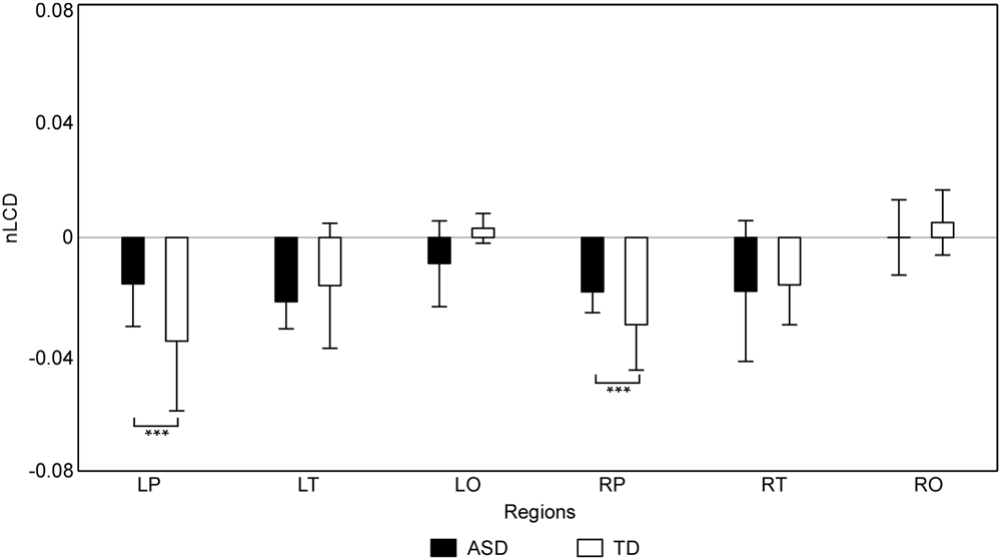
The statistical results of nLCDs. Means and standard deviations of nLCDs of six brain regions (i.e., LP, LT, LO, RP, RT and RO) for each group (ASD group: black bar; TD group: white bar), with error bars representing the standard deviations. Group differences marked by *** are corresponding to the significance level 0.001 (FDR-corrected).

The two-way ANOVA conducted on the nLCDs of six brain regions (i.e., LP-nLCD, LT-nLCD, LO-nLCD, RP-nLCD, RT-nLCD and RO-nLCD) revealed that the main effect of brain region and the interaction between brain region and group were significant (*F* (5, 110) = 11.41, *p* < 0.001, and *F* (5, 110) = 2.99, *p* < 0.05 respectively). Further analysis showed that (1) the nLCDs of bilateral prefrontal cortices and bilateral temporal lobes were significantly smaller than those of bilateral occipital lobes; (2) as has been shown in Fig. 2, the LP-nLCD and RP-nLCD of ASD group were significantly larger than those of TD group.

The one sample t-tests on nLCD of each brain region and each group found that the nLCDs of bilateral prefrontal cortices and bilateral temporal lobes (i.e., LP-nLCD, LT-nLCD, RP-nLCD and RT-nLCD) for both groups were significantly smaller than zero, meaning that the oxy-Hb signals in these regions could reduce the spatial complexity, thus increase the global functional connectivity level between the scalp channels for both groups.

### Variability of normalized spatial complexity

For the vnGSC, the independent samples t-tests did not detect any significant group effect (*ps* > 0.05), no matter which window length was selected.

For the vnRSCs of six brain regions (i.e., LP-vnRSC, LT-vnRSC, LO-vnRSC, RP-vnRSC, RT-vnRSC and RO-vnRSC), the main effect of brain region was significant (*F*s > 10, *ps < *0.001), no matter which window length was selected. The post hoc analysis revealed that the vnRSCs of bilateral prefrontal cortices and bilateral temporal lobes were significantly smaller than those of bilateral occipital lobes. The main effect of group was not significant for all the window lengths (*F*s < 1, *ps* > 0.05). However, the interaction effect was significant, when the window length was 70 sec (*F* (5, 110) = 3.31, *p* < 0.01). Further analysis revealed that the LO-vnRSC was significantly larger in ASD group, when the window length was 70 sec.

**Figure 3:**
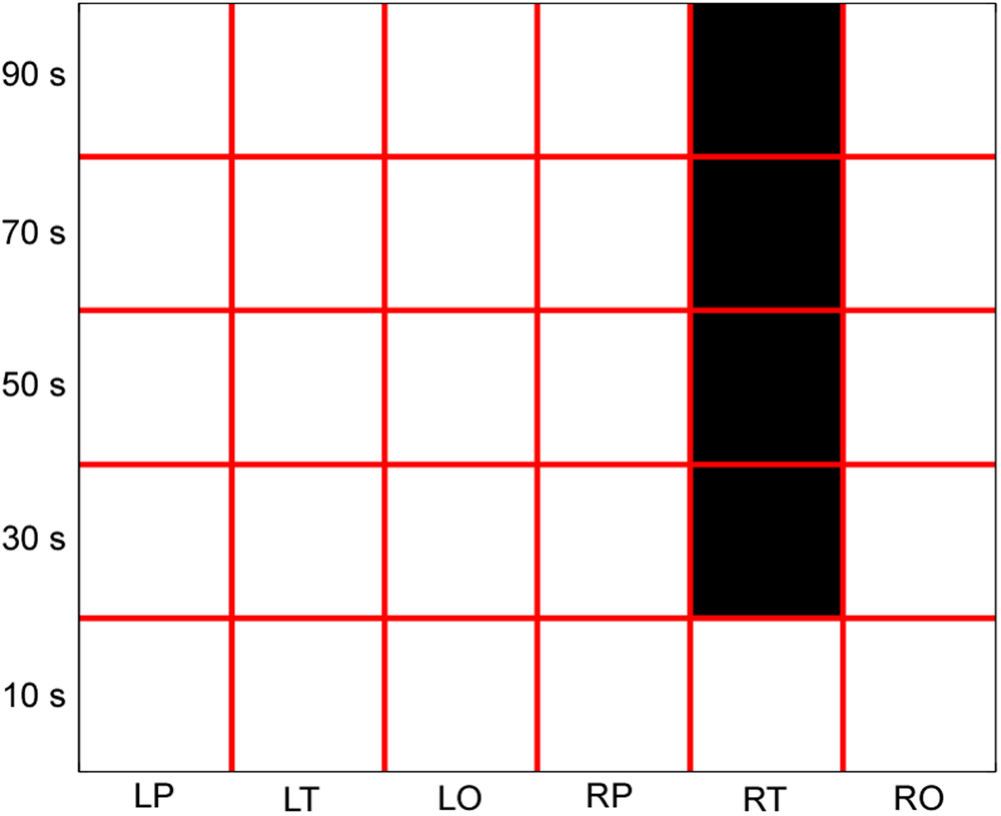
The statistical results of vnLCD of each brain region (i.e., LP, LT, LO, RP, RT and RO) and each window length (10 sec, 30 sec, 50 sec, 70 sec and 90 sec). For each window length, the brain regions with significant group difference (ASD > TD) are masked in black.

For vnLCD of the six regions (i.e., LP-vnLCD, LT-vnLCD, LO-vnLCD, RP-vnLCD, RT-vnLCD and RO-vnLCD), we found that the main effect of brain region was not significant (*F*s < 2, *ps* > 0.05), no matter which window length was selected. Moreover, the interaction between the two independent variables was significant, when the window length was 30 sec, 50 sec, 70 sec or 90 sec (*F*s > 2, *ps < *0.01). The simple effect analysis found that the RT-vnLCD of ASD group was significantly larger than that of TD group (*p* < 0.01), when the window length was 30 sec, 50 sec, 70 sec or 90 sec (Fig. 3). However, the main effects of group were not significant for all the window lengths (*F*s < 1, *ps* > 0.05).

## Discussion

In the present study, we proposed to apply PCA and normalized entropy to effectively estimate normalized spatial complexity of neural signals, which could reflect the global functional connectivity of brain signals and overcome the limitations inherent in omega complexity. The advantages of the presented approach were demonstrated through fNIRS-based ASD research. The results showed that: (1) the time-averaged nRSC of bilateral prefrontal cortices (LP-nRSC and RP-nRSC) and the time-averaged nLCD of bilateral prefrontal cortices (LP-nLCD and RP-nLCD) in ASD group were significantly higher than those in TD group; (2) the time-averaged nRSCs of bilateral prefrontal cortices were significantly smaller than those of bilateral temporal lobes, and the time-averaged nRSCs of bilateral temporal lobes were significantly smaller than those of bilateral occipital lobes; (3) the time-averaged nLCDs of bilateral prefrontal cortices and bilateral temporal lobes were significantly smaller than zero and those of bilateral occipital lobes; (4) the vnRSCs of bilateral prefrontal cortices and bilateral temporal lobes were significantly smaller than those of bilateral occipital lobes; (5) the vnLCD of right temporal lobe (RT-vnLCD) of ASD group was significantly larger than that of TD group, when the window length was 30 sec, 50 sec, 70 sec or 90 sec. These results suggest that the proposed normalized spatial complexity could overcome the limitations inherent in omega complexity, and highlight the crucial role of the prefrontal cortex and right temporal lobe in autism.

### The methodological advantages of the present approach

Cognition and consciousness are believed to emerge from activity of spatially remote brain areas coordinated by functional connectivity. Several approaches have been proposed in order to understand the organization of brain networks^2,4,11,33^. Among these approaches, the spatial complexity analysis of neural signals could indicate the global functional connectivity level across distinct ROIs^11^. Moreover, we could evaluate the contribution of each brain region to the global functional connectivity level using this approach. In this aspect, the omega complexity, proposed by Wackermann (1996), could be considered as an indicator of spatial complexity. Although the omega complexity has been widely used in related studies, an important limitation exists, i.e., the measures in omega complexity analysis are sensitive to the number of ROIs defined. Here, an alternative approach based on PCA and the normalized entropy was proposed to evaluate normalized spatial complexity of neural signals. Moreover, it’s well known that the brain networks are transient and dynamic, established on the timescale of milliseconds^2,34^. Thus, the current study not only calculated time-averaged normalized spatial complexity metrics, but also estimated the variability of normalized spatial complexity metrics using the sliding-window approach.

In our study, the number of channels in six brain regions (i.e., LP, LT, LO, RP, RT and RO) were 10, 8, 4, 10, 8, and 4. Since the metrics in omega complexity could be significantly influenced by the number of channels, it’s natural that (1) the time-averaged and variability of regional spatial complexities of bilateral occipital lobes were significantly smaller than those of bilateral prefrontal cortices and bilateral temporal lobes; (2) the time-averaged LCDs derived from traditional omega complexity of most brain regions were significantly larger than zero (see Supplementary Materials). Moreover, these time-averaged LCDs did not show any significant region difference (see Supplementary Materials). All these results indicate that metrics in traditional omega complexity would distort the relative spatial complexity level between brain regions and the contribution of each brain region to the global spatial complexity, which can be correctly detected by the normalized spatial complexity proposed here. Moreover, using normalized spatial complexity, we found that the vnLCD of right temporal lobe (RT-vnLCD) of ASD group was significantly larger than that of TD group (*p* < 0.01), when the window length was 30 sec, 50 sec, 70 sec or 90 sec. However, using traditional omega complexity, this group difference could only be found when the window length was 30 sec. This result suggest that the metrics derived from normalized spatial complexity may be more sensitive to those derived from traditional omega complexity, when we were testing the group difference between young children with ASD and age-, gender-matched TD children.

Using traditional functional connectivity, researchers could investigate the functional connectivity level between two ROIs using the ROI-based approach^35^, investigate the functional connectivity level within certain large-scale neural networks using the ICA- or seed-based approach^5,36^, or investigate the topographical organization using graph theory based network analysis^37^. However, all these approaches could not probe into the time-averaged or time-varying contribution of each brain region to the global functional connectivity, which could be estimated through nLCD and vnLCD proposed in this study. Using traditional functional connectivity, our group has found that the functional connectivity between the right prefrontal cortex and other brain regions (e.g., the left prefrontal cortex and the bilateral temporal lobe) were significantly weaker in young children with ASD, compared with age- and gender-matched TD children^32^. The results in the current study have extended previous findings by showing that (1) the nRSC and nLCD of bilateral prefrontal cortices in ASD group were significantly larger than those in TD group, and (2) the vnLCD of right temporal lobe of ASD group were significantly larger than that of TD group.

### Accordant conclusion on the pathological mechanisms in ASD

ASD is a developmental disability that can cause significant social, communication and behavioral challenges. A series of theories have been proposed to explain the underpinning neurobiological mechanisms of ASD^27,38,39^. A wealth of studies support that the ASD is closely associated to the aberrant neural connectivity in autistic persons’ brains^27^. For instance, using resting-state fMRI and Knowledge based functional connectivity Enrichment Analysis, Cheng *et al*. (2017) found that functional connectivity decreased at the network circuit level in patients with ASD compared with healthy controls in networks involving the orbitofrontal cortex, anterior cingulate cortex, middle temporal gyrus cortex, and the precuneus, in networks that are implicated in the sense of self, face processing, and theory of mind^25^. Using fNIRS, which is a neuroimaging technique more suitable for children research, significantly weaker lobe-level inter-region connections were uncovered in the right prefrontal cortex in young children with ASD when compared with healthy age- and gender-matched children, including its linkages with the left prefrontal cortex and the bilateral temporal cortex^32^.

Compared to traditional functional connectivity analysis, the nLCD provided a direct index to estimate the contribution of activities of specific brain region to the global functional connectivity of all regions. Here, the normalized local complexity differentials of bilateral prefrontal cortices (LP-nLCD and RP-nLCD) in ASD group were significantly higher than those in TD group. In addition, the LP-nLCD and RP-nLCD of both groups were significantly smaller than zero. It suggested that although the oxy-Hb signals in the bilateral prefrontal cortices could increase the global functional connectivity level between all brain areas for both groups, the contribution of bilateral prefrontal cortices was significantly reduced in ASD group. These results were highly consistent with Li & Yu (2016), although they failed to uncover that the left prefrontal cortex also made a major contribution to the hypo-connectivity of young children with ASD using traditional approaches.

### Novel findings on the pathological mechanisms in ASD

In the current study, the normalized regional spatial complexity of bilateral prefrontal cortices in ASD group was significantly higher than that in TD group, which suggested the global functional connectivity level was significantly lower in bilateral prefrontal cortices of ASD children. Using traditional functional connectivity analysis, Li & Yu (2016) failed to detect this effect. Previous studies found that the prefrontal cortex played a hub role in information integration^40^. Moreover, the prefrontal cortex abnormality was found to be central in autism and its abnormality could contribute to the social and non-social dysfunction in individuals’ behavior development^26,32^. Thus, the results in the current study were consistent with these previous findings, and highlighted that the prefrontal cortex made a major contribution to the hypo-connectivity of young children with ASD.

Moreover, we found that the nLCDs of bilateral prefrontal cortices and bilateral temporal lobes (i.e., LP-nLCD, LT-nLCD, RP-nLCD and RT-nLCD) for both groups were significantly smaller than zero, whereas the nLCDs of occipital lobe for both hemispheres and both groups were not significantly different from zero, which means that the oxy-Hb signals in prefrontal cortex and temporal lobe, not those in occipital lobe, could reduce the spatial complexity of data (i.e., increase the global functional connectivity level between the scalp channels) for both groups. Traditional functional connectivity analysis and omega complexity analysis failed to detect this effect (see Supplementary Materials).

Analysis on the variability of normalized spatial complexity metrics revealed that the variability of the normalized local complexity differential in right temporal lobe (RT-vnLCD) in ASD group was significantly higher than that in TD group, which suggested that the contribution of right temporal lobe to the global functional connectivity in ASD group may vary more dramatic than that in TD group during fNIRS recording. The right posterior superior temporal sulcus (pSTS), which is an important part of the right temporal lobe, is crucial for social cognition and is implicated in several steps of social interactions^41,42^. Many brain imaging studies have found anatomical and functional right pSTS abnormalities in ASD, which involves decreased gray matter concentration, rest hypoperfusion and abnormal activation patterns during social cognition tasks^43,44^. Another region of right temporal lobe that is of special relevance to autism is the “fusiform face area”, which is engaged in human face processing^45,46^. In the current study, the contribution of right temporal lobe to the global functional connectivity during fNIRS recording in ASD group was found vary more dramatic than that in TD group, which may significantly reduce the efficiency in social information processing. Thus, the increased RT-vnLCD may contribute to the social interaction impairment in children with ASD, which has not been found using traditional approaches.

### Methodological Considerations

Firstly, although the value of the proposed approach was validated using fNIRS signals from ASD studies, this novel technique could also be applied to the neural signals of other neuroimaging techniques, such as EEG, MEG and fMRI. However, it should be mentioned that due to the distinct properties of these neuroimaging signals, some additional steps may be needed. For example, for EEG/MEG signals, researchers need to extract the activities of each frequency band (e.g., delta, theta, alpha, beta and gamma band) before conducting normalized spatial complexity analysis. In the future studies, we need to study normalized spatial complexity of neural signals of children with ASD using other neuroimaging techniques, which may produce similar results. Moreover, since numerous studied have proved that age could modulate the brain structures and functions of patients with ASD^47,48^, it’s very interesting to investigate the effect of age of patients with ASD on normalized spatial complexity metrics. In the future studies, we should investigate this question.

Secondly, the approach proposed here was suggested to estimate the spatial complexity of multiple time series. There are many measures (e.g., entropies, fractal dimensions, and Lyapunov exponents), which could assess the complexity in the time dimension or the complexity of a dynamical time series^49–51^. Among all these measures, the permutation entropy (PE) is conceptually simple, computationally efficient and artifact resistant, which is more suitable for application of complexity analysis to fNIRS signals^52^. These measures are not completely independent from the proposed normalized spatial complexity metrics. In the future study, the PE could be used to quantify the temporal complexity of dynamical time series of normalized spatial complexity metrics.

Lastly, more valuable results may be produced, if the approach proposed here is applied combining with other methods. For example, Yan *et al*. (2015) proposed that the global synchronization index (GSI), which assess synchronization in multivariate neural signals, could be calculated using the phase correlation matrix derived from the minimum variance distortion-less response magnitude squared coherence (MVDR-MSC) method^53^. This approach is closely related with our normalized spatial complexity analysis. However, the metrics in our approach are based on covariance matrix or PCA decomposition instead of the phase correlation matrix. Moreover, the approach in Yan *et al*. (2015) did not explicitly give any information regarding how to compute the contribution of activities of specific ROI to the global synchronization of all ROIs and how to estimate the temporal variability of global synchronization. In the future, researchers could combine these two approaches, which may yield more valuable results.

## Conclusion

Here, normalized spatial complexity, combining PCA and the normalized entropy, was introduced, which was expected to overcome the limitations inherent in traditional omega complexity. This approach was verified by the fNIRS data from a previous published ASD research. By this new method, several conclusions consistent with traditional approaches on the pathological mechanisms of ASD were found, i.e., the prefrontal cortex made a major contribution to the hypo-connectivity of young children with ASD. Moreover, some novel findings on the pathological mechanisms of ASD were also detected, which could not be successfully detected via traditional approaches. These results confirmed the value of this novel approach, and extended the methodology system of functional connectivity. This novel technique could be applied to the neural signal of other neuroimaging techniques and other neurological and cognitive conditions.

## Methods

### Participants and Experimental procedures

Sixteen children with ASD were recruited for the current study. Four of these children were excluded from the analysis due to failure of fNIRS data collection. Therefore, 12 children with ASD aged 6.1 ± 1.1 years (mean ± SD, range: 4~9 years) were used in data analysis. Besides, 12 age- and gender-matched TD children recruited in this study. All children with ASD came from a local special school (NANJING MINGXIN Intelligence-Promoting School, NJMXIPS) and were diagnosed with ASD in the past two years before data recording by clinicians of a local hospital (NANJING Brain Hospital) using standard procedures. All these children achieved the diagnostic criteria of autism depending on the 4th edition of Diagnostic and Statistical Manual of Mental Disorders (DSM-4). Parents of each participant were requested to report their child’s behavior using the Autism Behavior Checklist (ABC)^22^. Besides, all these ASD children were identified as high-functioning autism according to their teachers’ reports. None of the children in the TD group had a history of psychiatric or neurological disorder. Parents of all participants signed an informed consent for the present experiment, which was approved by the local Institutional Review Board of Southeast University and conducted in accordance with the principles of the Declaration of Helsinki.

During collecting concentration changes of oxy-hemoglobin (oxy-Hb) and deoxy-hemoglobin (deoxy-Hb), all these children were asked to wear customized fNIRS cap and watch a popular Chinese cartoon, since some of the ASD children were usually irritable in the fNIRS scanning. The parent or teacher of each child was asked to hold this child, so as to make sure he/she would feel safe during the experiment and to confirm that he/she was concentrating on the video program. Besides, a camera was used to record the behaviors of these children during fNIRS recording so that the corrupted data caused by large head/body movements or other unexpected behaviors could be identified and removed in data preprocessing. It takes about 14 min to complete the fNIRS scanning.

More information about the participants and experimental procedures could be seen in our previous study^32^.

### fNIRS data acquisition

**Figure 4:**
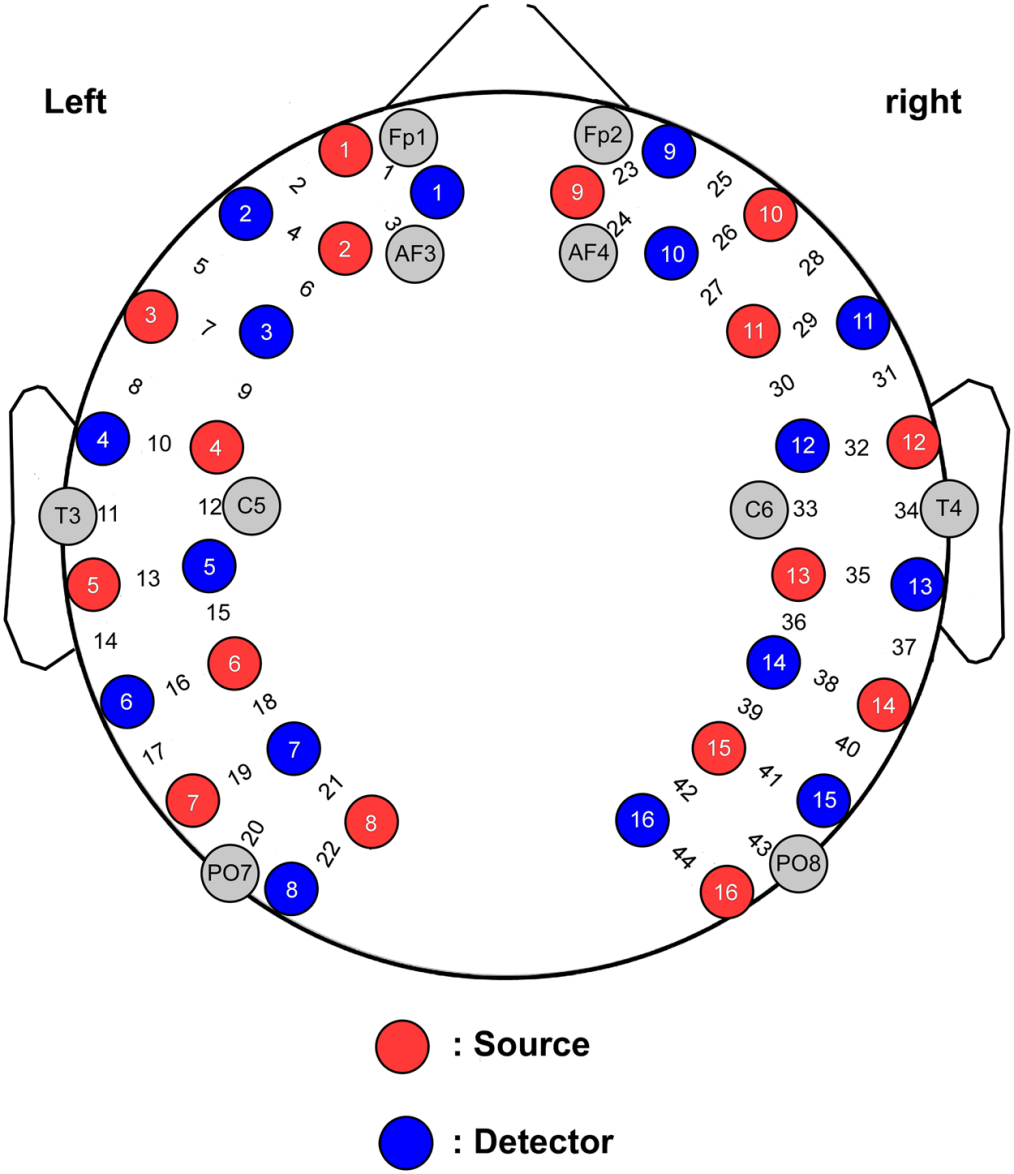
Schematic of the probe/channel configurations on the head. Red and blue circles indicate the source emitters and the photon detectors, respectively. The 44 measurement channels are located between emitter/detector pairs.

A multichannel fNIRS system, LABNIRS (Shimadzu Corporation, Kyoto, Japan) with three wavelengths (i.e., 780, 805 and 830 nm), was applied to record the concentration changes in oxy-Hb and deoxy-Hb. A pair of emitter probe and neighboring detector probe, which were plugged into a customized cap (distance between adjacent probes was 30 mm), formed one channel. A total of 16 emitter probes and 16 detector probes were placed on the scalp, resulting in 44 channels. The probe/channel configurations on the head are shown in Fig. 4. The scanning rate of this fNIRS system was 27 ms, resulting in a sampling rate of about 37 Hz. The recorded changes in optical density were converted into concentration changes of oxy-Hb and deoxy-Hb using the modified Beer-Lambert law^54^.

### Data preprocessing

The fNIRS signal preprocessing was consisted of following steps:

Firstly, data segments contaminated by large head movements, unexpected behaviors and sharp changes in oxy-Hb and deoxy-Hb signals were identified and removed.

Secondly, a band-pass filtering (0.009–0.08 Hz) was performed on the oxy-Hb and deoxy-Hb signals^55,56^. Such strict noise control procedures could validly reduce the influence of typical noise components in fNIRS signals.

Lastly, only the time series of the hemoglobin concentration signals between 1 to 8 min were included in the following analysis, in order to eliminate the influence of signal length on group difference.

Compared to concentration changes of deoxy-Hb, the oxy-Hb has been found to be a more sensitive metric of regional cerebral blood flow and provided a more robust positive correlation with the BOLD signal^55^, thus the oxy-Hb concentration was chosen to compute normalized spatial complexity metrics of participants in the current study.

### Estimation of normalized spatial complexity metrics

In the current study, two kinds of normalized spatial complexity metrics were estimated. Firstly, time-averaged, static normalized spatial complexity metrics were calculated, which were normalized global spatial complexity (nGSC) of all 44 channels, normalized regional spatial complexities (nRSCs) and normalized local complexity differentials (nLCDs) of left prefrontal cortex (LP, channels 1~10), right prefrontal cortex (RP, channels 23~32), left temporal lobe (LT, channels 11~18), right temporal lobe (RT, channels 33~40), left occipital lobe (LO, channels 19~22) and right occipital lobe (RO, channels 41~44). Secondly, the characteristics of temporal variation in normalized spatial complexity were quantified by the variability of normalized global spatial complexity (vnGSC) of all 44 channels, the variability of normalized regional spatial complexity (vnRSC) and the variability of normalized local complexity differential (vnLCD) of each brain region. The steps of computing these two kinds of metrics are as follows:

### Time-averaged normalized spatial complexity metrics computation

**Figure 5:**
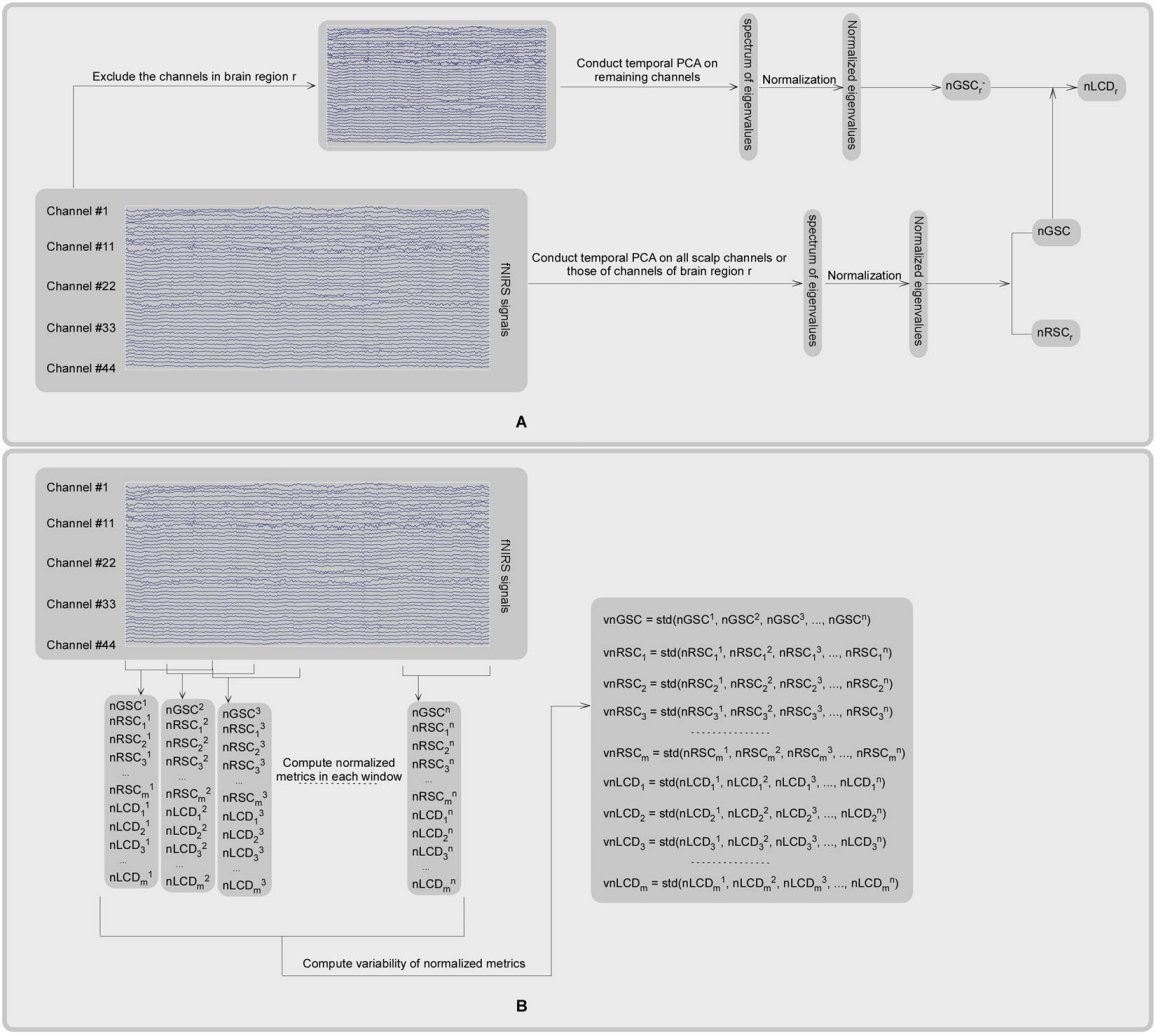
The procedure of time-averaged normalized spatial complexity metrics computation (panel A) and variability of normalized spatial complexity metrics computation (panel B). Panel A: In order to compute time-averaged nGSC and nRSC of region r (i.e., nRSC_r_), temporal PCA was conducted on the fNIRS signals of all scalp channels and those of region r respectively, which resulted in spectrum of eigenvalues. Then these eigenvalues were normalized to unit sum (see Equation [1]). Lastly, the nGSC or nRSC_r_ was computed using the Equation (2). During the computation of nLCD of region r (i.e., nLCD_r_), the normalized global spatial complexity when excluding the channels in region r from the analysis (i.e., nGSC_r_^−^) was computed firstly. Then the nLCD_r_ was computed as the difference between nGSC and nGSC_r_^−^ (see Equation [3]). Panel B: Firstly, the entire fNIRS signal was divided into a dozen of overlapping time windows. Then, the nGSC, the nRSC and nLCD of each brain region within each time window were computed using the procedure illustrated in the panel A. Lastly, the standard deviations of these normalized spatial complexity metrics across these time windows were computed, which were used as metrics that quantify the vnGSC, the vnRSC and the vnLCD of each brain region. In this panel, m and n denoted the number of brain regions (6 in this case) and the number of time windows respectively. Note that, although this procedure was initially designed for fNIRS signals, it could be easily applied to other kinds of brain signals (e.g., EEG, MEG, and fMRI).

The nGSC of oxy-Hb signal for each participant was estimated as following (see panel A of Fig. 5).

Firstly, temporal principal component analysis (PCA) was conducted on the oxy-Hb signal, which yielded m principal components (m is the number of channels across the scalp, i.e., 44 in this case) and spectrum of eigenvalues.

Secondly, to assess the relative contribution of each principal component to the total variance, the eigenvalues of principal components were normalized to unit sum. The normalized eigenvalue of the i th principal component was calculated as

1 $${\lambda }_{i}^{\text{'}}={\lambda }_{i}/\sum _{i=1}^{m}{\lambda }_{i}^{\text{'}}$$

where m was the number of principal components or scalp channels (44 in this case), *λ*_*i*_ and *λ*′_*i*_ represented the eigenvalue and the normalized eigenvalue of the *i* th principal component respectively. Lastly, the nGSC, defined as the normalized entropy of normalized eigenvalues, was computed using the following equation:

2 $${\rm{nGSC}}=-\,\frac{{\sum }_{i=1}^{m}{\lambda }_{i}^{\text{'}}\,\mathrm{log}\,{\lambda }_{i}^{\text{'}}}{\mathrm{log}\,m}$$

The nGSC computed above attains values from the interval 0 to 1. The lowest value nGSC = 0 means the oxy-Hb signals of all scalp channels are consisted of exactly one principal component or spatial mode, and a maximum global functional connectivity between all the scalp channels is detected. The highest value nGSC = 1 indicates the total data variance is uniformly distributed across all m principal components, and a maximum spatial complexity or a lowest global functional connectivity is found.

The procedure of the computation of nRSCs of six brain regions (i.e., LP, LT, LO, RP, RT and RO) were similar as that of nGSC, except that only the channels in that region were included in PCA decomposition (see panel A of Fig. 5).

In order to evaluate regional contributions to nGSC, the nLCD of each brain region, which was defined as the variation of nGSC obtained by excluding the channels in that region from the nGSC evaluation, was estimated for each participant (see panel A of Fig. 5). The nLCD of region r is defined as:

3 $$nLC{D}_{r}=nGSC-nGS{C}_{r}^{-}$$

where nGSC is the normalized global spatial complexity computed using all the 44 scalp channels, nGSC_r_^−^ is the normalized global spatial complexity computed when excluding the channels in region r from the analysis. nLCD_r_ < 0 means that the oxy-Hb signals in region r could reduce the spatial complexity of data, thus increase the global functional connectivity level between the scalp channels. However, nLCD_r_ > 0 means that the oxy-Hb signals in region r could increase the spatial complexity of data, thus reduce the global functional connectivity level between the scalp channels.

### The computation of variability of normalized spatial complexity metrics

For each participant, the dynamic characteristics of normalized spatial complexity metrics of oxy-Hb signals were estimated using the sliding-window approach, in which the entire oxy-Hb time series was divided into a dozen of overlapping time windows (see panel B of Fig. 5). The overlapping between adjacent windows is 50%. Note that there are few researches studying the time-varying nature of functional connectivity in fNIRS signals^57^. In order to avoid arbitrary selection of window length, several window lengths (i.e., 10, 30, 50, 70 and 90 sec) were used. Then, the normalized spatial complexity metrics (i.e., the nGSC, the nRSCs and the nLCDs of the above six regions) within each time window were computed using the procedure illustrated in the above section. Lastly, the standard deviations of these normalized spatial complexity metrics across these time windows were computed, which were used as metrics that quantify the vnGSC, the vnRSCs and the vnLCDs of the above six regions (see panel B of Fig. 5).

### Statistical tests

For the nGSC and the vnGSC of each window length, independent samples t-test was conducted to test group effect.

For the nRSCs, the nLCDs, and the vnRSCs and vnLCDs of each window length, a two-way ANOVA was performed, respectively. The two independent variables are brain region (LP, LT, LO, RP, RT and RO) and group (ASD and TD).

Moreover, for nLCDs of six brain regions in two groups, one sample t-tests were performed in order to statistically test whether the nLCD of each brain region and each group was significant different from zero.

In order to control multiple comparisons, the significance level (*p* value) was corrected using false discovery rate (FDR) procedure^58^.

### Availability of Data and Materials

The datasets generated and/or analysed during the current study are not publicly available but are available from the corresponding author on reasonable request.

## Electronic supplementary material

Supplementary Information
