# Supplementary material for: Normalized spatial complexity analysis of neural signals
Source: Sci Rep. 2018 May 21;8:7912. doi: 10.1038/s41598-018-26329-0 (PMC5962588; doi:10.1038/s41598-018-26329-0)
Supplement: Supplementary file 1 — Supplementary Information [file 41598_2018_26329_MOESM1_ESM.doc]

**Normalized spatial complexity analysis of neural signals**

Huibin Jia^1^, Yanwei Li^2,1^, Dongchuan Yu^1^*

Huibin Jia and Yanwei Li contributed equally to this work.

^1^ Key Laboratory of Child Development and Learning Science of Ministry of Education, School of Biological Sciences & Medical Engineering, Southeast University, Nanjing, Jiangsu, China.

^2^ College of Preschool Education, Nanjing Xiaozhuang University, Nanjing, Jiangsu, China.

***Corresponding author:**

E-mail address: dcyu@seu.edu.cn (Dongchuan Yu)

Current address: Room 323, Liwenzheng Building, Southeast University, No. 2, Sipailou, Xuanwu District, Nanjing, China, 210096

**The estimation and statistical tests on omega complexity metrics**

Similar to the estimation on normalized spatial complexity metrics, time-averaged omega complexity metrics (i.e., the GSC, the RSCs and LCDs of six brain regions) and temporal variation of omega complexity metrics (i.e., the vGSC, the vRSCs and vLCDs of six brain regions) were computed. The estimation of time-averaged omega complexity metrics and omega complexity metrics in each time window (i.e., single-window omega complexity metrics) were based on temporal PCA and Shannon entropy, and were calculated as the Shannon entropy of normalized eigenvalues. Thus, the difference between the omega complexity analysis and normalized spatial complexity analysis when computing time-averaged/single-window omega complexity metrics occurs in the last step. For example, the GSC was calculated using the following equation:

The GSC computed above attains values from the interval 1 to m. The lowest value GSC = 1 means the oxy-Hb signals of all scalp channels are consisted of exactly one principal component or spatial mode, and a maximum global functional connectivity between all the scalp channels is detected. The highest value GSC = m indicates the total data variance is uniformly distributed across all m principal components, and a maximum spatial complexity or a lowest global functional connectivity is found.

The statistical tests on omega complexity metrics were the same as those on normalized spatial complexity metrics.

**Results of statistical tests on omega complexity metrics**

*Time-averaged omega complexity metrics*

Significant group difference between the two groups was detected for GSC (t = 2.43, df = 22, p = 0.02 < 0.05). The GSC of ASD group (8.54 ± 1.63) was significantly larger than that of TD group (7.30 ± 0.70).

For the RSCs of six brain regions (i.e., LP-RSC, LT-RSC, LO-RSC, RP-RSC, RT-RSC and RO-RSC), the main effect of brain regions and the interaction between brain region and group were significant (F (5, 110) = 31.02, p < 0.001, and F (5, 110) = 4.88, p < 0.01 respectively). Further analysis showed that (1) the RSCs of bilateral occipital lobes were significantly smaller than those of bilateral prefrontal cortices and bilateral temporal lobes; (2) the LP-RSC and RP-RSC of ASD group was significantly larger than those of TD group. The main effect of group was significant, F (1, 22) = 5.52, p < 0.05.

The two-way ANOVA conducted on the LCDs of six brain regions (i.e., LP-LCD, LT-LCD, LO-LCD, RP-LCD, RT-LCD and RO-LCD) revealed that the main effect of brain region was not significant, F (5, 110) = 0.70, p > 0.05. The interaction between brain region and group was significant, F (5, 110) = 3.93, p < 0.01. Simple effect analysis found that the LP-LCD and RP-LCD of the ASD group was significantly larger than those of TD group. The main effect of group was also significant, F (1, 22) = 5.59, p < 0.05. The LCD of ASD group was significantly larger than that of TD group.

The one sample t-tests on LCDs of each brain region and each group found that: (1) For ASD group, the LP-LCD, LT-LCD, RP-LCD and RO-LCD were significantly larger than zero; (2) For TD group, the LO-LCD, RT-LCD and RO-LCD were significantly larger than zero.

*Variability of omega complexity metrics*

For the vGSC, the independent samples t-tests did not detect any significant group effect (ps > 0.05), no matter which window length was selected.

For the vRSCs of six brain regions (i.e., LP-vRSC, LT-vRSC, LO-vRSC, RP-vRSC, RT-vRSC and RO-vRSC), the main effect of brain region was significant (Fs > 10, ps < 0.001), if the window length was 30 sec, 50 sec, 70 sec or 90 sec. The post hoc analysis revealed that the vRSCs of bilateral occipital lobes were significantly smaller than those of bilateral prefrontal cortices and bilateral temporal lobes. However, the main effect of group and the interaction between the two factors were not significant for all the window lengths (Fs < 2, ps > 0.05).

For vLCD of the six regions (i.e., LP-vLCD, LT-vLCD, LO-vLCD, RP-vLCD, RT-vLCD and RO-vLCD), we found that the main effect of brain region was not significant (Fs < 2, ps > 0.05), no matter which window length was selected. Moreover, the interaction between the two independent variables was significant, when the window length was 30 sec, F (5, 280) = 2.39, p < 0.05. The simple effect analysis found that the RT-vLCD of ASD group was significantly larger than that of TD group (p < 0.05), when the window length was 30 sec. However, the main effect of group was not significant for all the window lengths (Fs < 1, ps > 0.05).
